# Impaired Resolution of Inflammation in Alzheimer’s Disease: A Review

**DOI:** 10.3389/fimmu.2017.01464

**Published:** 2017-11-06

**Authors:** Robert A. Whittington, Emmanuel Planel, Niccolò Terrando

**Affiliations:** ^1^Department of Anesthesiology, College of Physicians and Surgeons, Columbia University, New York, NY, United States; ^2^Faculté de Médecine, Département de Psychiatrie et Neurosciences, Université Laval, Québec City, QC, Canada; ^3^Centre de Recherche du CHU de Quebec, Centre Hospitalier de l’Université Laval, Neurosciences, Québec City, QC, Canada; ^4^Department of Anesthesiology, Duke University, Durham, NC, United States

**Keywords:** Alzheimer’s disease, resolution, inflammation, beta-amyloid, tau, resolvins, specialized pro-resolving lipid mediators, omega-3 fatty acids

## Abstract

Alzheimer’s disease (AD) remains the leading cause of dementia worldwide, and over the last several decades, the role of inflammation in the pathogenesis of this neurodegenerative disorder has been increasingly elucidated. The initiation of the acute inflammatory response is counterbalanced by an active process termed resolution. This process is designed to restore homeostasis and promote tissue healing by the activation of neutrophilic apoptosis, promotion of neutrophil clearance by macrophages, and increasing anti-inflammatory cytokine levels, while concurrently leading to a diminution in pro-inflammatory mediators. The switch from the initiation to the resolution phase of inflammation is initially characterized by increased production of arachidonic acid-derived pro-resolving lipoxins and decreases in pro-inflammatory prostaglandin and leukotriene levels, subsequently followed by increases in specialized pro-resolving lipid mediators derived from omega-3 fatty acids (ω-3 FAs). There is mounting evidence that in AD, the resolution of inflammation is impaired, resulting in chronic inflammation and the exacerbation of the AD-related pathology. In this review, we examine preclinical and clinical evidence supporting the hypothesis that AD is a neurodegenerative disorder where the impairment or failure of resolution contributes to the disease process. Moreover, we review the literature supporting the potential therapeutic role of ω-3 FAs and specialized pro-resolving lipid mediators in the management of the disease. Lastly, we highlight areas that could strengthen the association of failed resolution to AD and should, therefore, be the focus of future scientific investigations in this research field.

## Introduction

With an estimated global prevalence of 46.8 million affected individuals in 2015, Alzheimer’s disease (AD) remains the leading cause of dementia worldwide (1). The majority of new AD cases are sporadic and late-onset in nature, and without a major therapeutic breakthrough, it is estimated that the prevalence will quadruple by the middle of this century (2), ultimately reaching a global prevalence of approximately 131.5 million (1). The major neuropathological hallmarks of AD include extracellular senile plaques composed of aggregates of beta-amyloid (Aβ) protein (3), and intraneuronal neurofibrillary tangles made up of aggregated tau protein (4, 5). Although the German psychiatrist and neuropathologist Alois Alzheimer described the neuropathological hallmarks of AD over a century ago (6), the role of inflammation in the pathogenesis of the disease was not fully appreciated until several decades later.

Recent work has demonstrated that the termination of the acute inflammatory response is dependent on an active process termed “resolution” (7–9). In this review, we will review the scientific evidence linking the impairment or failure of resolution to AD, the specific neuropathological consequences of resolution failure in this neurodegenerative disorder, and the potential for the restoration of resolution to serve as a therapeutic target in AD.

## Neuroinflammation and AD

Neuroinflammation has been implicated in playing a key role in the pathogenesis of AD, with studies suggesting various mechanisms including astrocyte (10–13) and microglial activation (14–18), increases in pro-inflammatory molecules such as cytokines and chemokines [reviewed in Ref. (19)]. Moreover, there is evidence to suggest that the activation of endothelial cells of the neurovascular unit, oligodendrocytes, and even neurons may be involved [reviewed in Ref. (20)]. In AD patients, the role of neuroinflammation is clinically supported by PET studies demonstrating microglial activation (21–23), increased serum (24–26), and brain (27, 28) pro-inflammatory cytokine levels as well as the downregulation of anti-inflammatory molecules in postmortem brain tissue (29). Moreover, although the CNS was once considered an immunologically privileged site, it is now recognized that peripheral inflammation can exacerbate the inflammatory environment of the brain and contribute to chronic neuroinflammation and neurodegeneration (30, 31).

## Resolution of Inflammation: A Vital Process for the Restoration of Tissue Homeostasis

The initiation of the acute inflammatory phase, usually in response to trauma, infection, tissue injury, neoplasia, or other major homeostatic stressors, has been well characterized by the increased release of pro-inflammatory mediators such as prostaglandins and leukotrienes, leading to polymorphonuclear (PMN) leukocyte recruitment and monocyte-macrophage proliferation. It was initially believed that the acute response to inflammation passively dissipated over time; however, it has been more recently appreciated that acute inflammation is actually kept in homeostatic balance by resolution (32, 33), which ultimately results in the clearance of recruited granulocytes and a restoration of pre-activation immune profiles [reviewed in Ref. (7, 9)]. Failure of resolution has been associated with the development of chronic inflammation (34), which has been implicated in the pathogenesis of many diseases including asthma, periodontitis, rheumatoid arthritis, ulcerative colitis, multiple sclerosis, and AD [reviewed in Ref. (9)].

**Figure 1:**
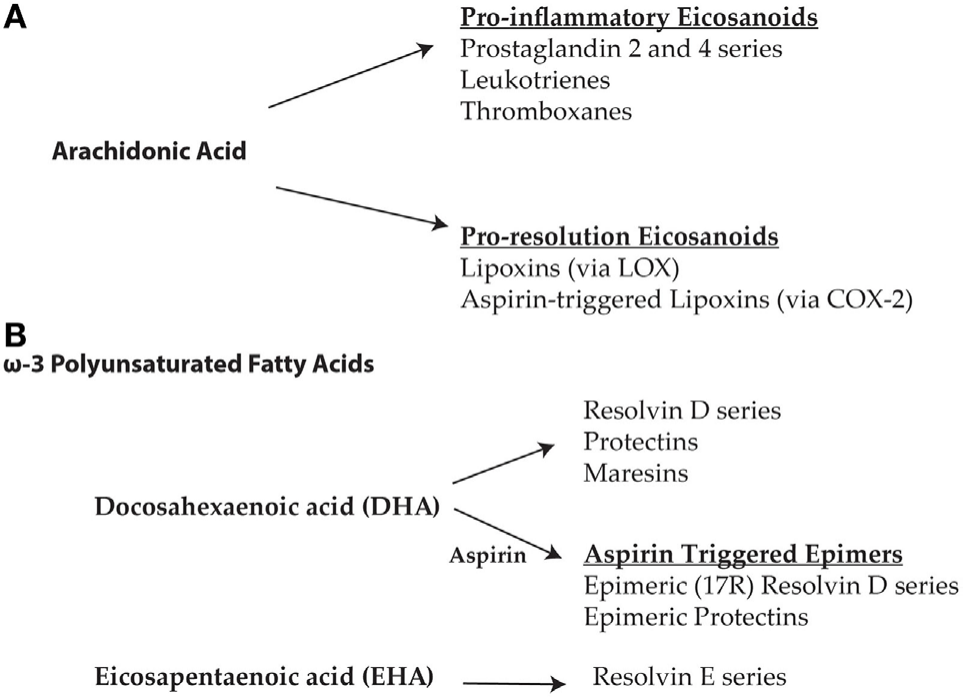
**(A)** The initiation phase of acute inflammation is characterized by an increase in the arachidonic acid-derived pro-inflammatory eicosanoids including prostaglandins, leukotrienes, and thromboxanes. Increases in prostaglandins eventually lead to a change in the biosynthesis of eicosanoids derived from arachidonic acid resulting in the formation of pro-resolution lipoxins via lipoxygenase-associated pathways. In the presence of aspirin, cyclooxygenase-2 is acetylated leading to the formation of the 15-epi-lipoxins (aspirin-triggered lipoxins). The aspirin-triggered isoforms are more resistant to degradation and hence more bioactive than native specialized pro-resolving mediators (SPMs). **(B)** Resolution is further propagated by the synthesis of SPMs derived from the omega-3 polyunsaturated fatty acids, docosahexaenoic acid (DHA), and eicosapentaenoic acid (EPA). SPMs derived from DHA include the resolvin D series, protectins, and maresins, as well as the aspirin-triggered epimeric (17R) forms of the resolvin D series and protectins. EPA derived SPMs include the resolvin E series.

The resolution of inflammation is initiated by a change in eicosanoid signaling that shifts from a pro-inflammatory to a pro-resolution, anti-inflammatory state, which is characterized by the biosynthesis of specific mediators. Sophisticated advances in lipidomics and metabolomics, spearheaded by seminal investigations by Serhan and his colleagues, have resulted in the identification and functional classification of these specialized pro-resolving mediators (SPMs) that drive resolution (7–9, 35–38). During the initiation phase of the acute inflammatory response, mediators derived from arachidonic acid become up-regulated and contribute to changes in vascular permeability and PMN leukocyte recruitment (39). However, the generation of these pro-inflammatory autacoids is eventually terminated by subsequent dynamic changes in prostaglandins E2 and D2 (39). This can be seen as a *switch* where increased levels of SPMs, including pro-resolving lipoxins [e.g., Lipoxin A4 (LXA4)], diminish inflammatory molecules such as prostaglandins, leukotrienes, and cytokines (Figure 1A). Pivotal to this *switch* is a class of lipid mediators, or SPMs, which are biosynthesized from the omega-3 fatty acids (ω-3 FAs), docosahexaenoic acid (DHA) and eicosapentaenoic acid (EPA), and regulate acute inflammation by promoting active resolution (Figure 1B). These SPMs include the resolvin D series compounds, protectins/neuroprotectins, and maresins, which are derived from DHA, as well as the resolvin E series compounds derived from EPA (Figure 1B) [reviewed in Ref. (34, 38, 40)]. The pro-resolving effects of the lipoxins and the ω-3 FA-derived SPMs collectively exert key features of resolution: suppression of PMNs, decrease vascular permeability, promotion of non-phlogistic monocyte recruitment, and increase macrophage-mediated clearance of apoptotic PMNs. However, during chronic inflammatory states, including neurodegeneration, this balance is disrupted and the predominance of arachidonic acid-derived pro-inflammatory lipid mediators contributes to the impaired resolution of inflammation and the persistence of a generalized pro-inflammatory state.

## Preclinical Studies Supporting Impaired Resolution in AD

### *In Vivo* Studies

Aging is associated with increased inflammation (41–43), and this, in itself, is of critical importance as it also is the major risk factor for the development of late-onset AD. Indeed, it has been recently demonstrated in BalbC mice that aging is associated with a delay in the resolution of inflammation. This is characterized by a greater increase and slower clearance of recruited PMNs following an acute inflammatory challenge, resulting in higher levels of pro-inflammatory cytokines and defective SPM production (44). Thus, given the impact that aging has on the development of AD, it is reasonable to hypothesize that age-related defects in resolution and “inflammaging” (45) may potentially contribute to the development and propagation of this neurodegenerative condition.

Over the last several years, there has been an increase in preclinical studies demonstrating that failed resolution plays a role in the development of AD pathology. Wang et al. demonstrated in senescence-accelerated mice-prone 8 (SAMP8), a murine model of accelerated aging that spontaneously exhibits β-amyloid (Aβ) overproduction, tau hyperphosphorylation, oxidative stress damage, and cognitive decline (46) that aging was indeed associated with a pro-inflammatory state (47). However, when compared with similarly aged 9-month-old senescent-accelerated mouse resistant 1 (SAMR1) mice, a mouse strain that displays normal aging without cognitive decline, SAMP8 mice did not exhibit any difference in LXA4 or resolvin D1 (RvD1) levels, despite a greater degree of inflammation in SAMP8 mice. Levels of ALX/FPR2 (*N*-formyl peptide receptor 2), the G protein-coupled receptor for Annexin-A1, LXA4, and RvD1, were also increased in SAMP8 mice but remained at levels similar to that of SAMR1 mice. This suggests that, despite the increased inflammation observed in SAMP8 mice, there is not a commensurate increase in SPMs. Hence, changes in ALX/FPR2 receptor density may actually be a compensatory response. Interestingly, in 9-month-old SAMP8 mice, these same investigators observed lower hippocampal levels of leukocyte-type 12-lipoxygenase (L12-LOX), the initial enzyme involved in the production of LXA4 and RvD1 from AA and DHA, respectively, when compared to similarly aged SAMR1 mice. Furthermore, L12-LOX co-localized with Aβ in the hippocampus. Interestingly, the lower L12-LOX levels positively correlated with increased levels of phosphorylated tau at the Ser^202^/Thr^205^ (AT8) phosphoepitope, suggesting that the impaired resolution response may impact the degree of Aβ and tau pathology that manifests with age in this strain.

Recent studies, utilizing lipoxin treatment in transgenic mice that develop AD-like pathology, have also provided compelling preclinical evidence supporting the role of impaired resolution in the development of AD pathology (48, 49). Lipoxins, particularly LXA4 and their aspirin-triggered (AT) carbon-15 (15R) epimers, are potent promoters of resolution by antagonizing pro-inflammatory mediators *via* activation of ALX/FPR2 receptors, resulting in decreases in leukocyte recruitment, NF-κB activation, superoxide generation, and longer-lasting effects on pro-inflammatory chemokine/cytokine production (35, 50–53). Aspirin has been shown to modulate lipoxin biosynthesis by yielding 15R epimerization products termed AT lipoxins, thus making it more resistant to inactivation and further promoting resolution signaling (52). In Tg2576 mice, which harbor the Swedish double mutation in human amyloid precursor protein leading to the rapid development of Aβ-related pathology, the administration of AT-LXA4 has been shown to reduce the activation of NF-κB and levels of pro-inflammatory cytokines as well as increase levels of the anti-inflammatory cytokine IL-10, ultimately resulting in an alternative microglial phenotype (48). This subsequent change in microglial phenotype was associated with improved phagocytosis, increased Aβ clearance, decreased synaptotoxicity, as well as cognitive improvement in the Tg2576 mice. More recently, Dunn et al. demonstrated in triple transgenic AD (3xTg-AD) mice, a transgenic mouse strain that expresses the Aβ-processing related mutations APP_SWE_ and PS1_M146V_ as well as mutant, pro-aggregant tau (tau_P301L_), thus respectively developing accelerated senile plaque and neurofibrillary tangle pathology, that brain LXA4 levels significantly decreased with age. This decrease in LXA4 levels was more pronounced in the 3xTg-AD mice than in non-transgenic mice; moreover, an 8-week treatment with AT-LXA4 reduced cognitive impairment, Aβ levels, and tau phosphorylation (49). Interestingly, the reduction in brain tau phosphorylation was mediated by decreased activation of the tau-related kinases, GSK-3β and p38 MAPK. Taken together, these preclinical studies support the concept that increased lipoxin signaling restores resolution physiology that, in turn, may serve as therapeutic means for attenuating Aβ and tau pathology as well as cognitive decline.

There is now growing interest as to whether the resolvins can actually attenuate AD pathology and modulate cognitive deficits, especially as the potentially neuroprotective effects of SPMs are increasingly elucidated. Using a mouse model of postoperative cognitive dysfunction following orthopedic surgery, acute administration of AT RvD1 prevented neuronal dysfunction and cognitive impairment by regulating long-term potentiation, and astrocyte activation (54). Using this same murine neuroinflammation model, we are currently conducting studies to determine whether the resolvins or other SPMs can attenuate changes in tau pathology following surgery-induced neuroinflammation. Recently, DHA-derived neuroprotectin D1 (NPD-1) has been observed to exert potent neuroprotective effects in multiple models of CNS injury and neurodegeneration by modulating synaptic plasticity, dendritic spine morphology, and microglia activation [reviewed in Ref. (55)]. Other SPMs, including resolvin E1 (RvE1), have also been implicated in the regulation of excitotoxic signaling, synaptic transmission, and neuroinflammation in models of inflammatory and postoperative pain (56). Although the exact mechanisms of resolvins on neuron–glia interactions have been only partly elucidated, SPMs may provide novel strategies to modulate and possibly prevent the onset and progression of neurodegenerative conditions including AD.

### *In Vitro* Studies

Human CHME3 microglia incubated with Aβ_42_, when compared with lipopolysaccharide (LPS) stimulation, have been recently observed to manifest decreased phosphorylation at serine 523 of 5-lipoxygenase (57), a key enzyme involved in the regulation of leukotriene and lipoxin production. This decreased phosphorylation at serine 523 resulted in increased leukotriene production and decreased lipoxin formation, which are changes consistent with disrupted resolution. Interestingly, Aβ_42_ or LPS did not alter the levels of LXA4 or RvD1 in the cell culture medium; furthermore, in contrast to the increase observed with LPS, Aβ_42_ had no effect on receptor levels of ALX/FPR2, the receptor for LXA4 and RvD1. Therefore, when compared to the pro-inflammatory stimulus LPS, it appears that Aβ_42_ is associated with changes that are consistent with impaired resolution and the potential propagation of a chronic inflammatory state.

Zhu et al. more recently demonstrated that LXA4, maresin 1, protectin DX, and RvD1 decreased staurosporine-induced apoptosis in human SH-SY5Y neuroblastoma cells (58). Furthermore, maresin 1 was observed to be particularly protective in terms of Aβ-related pathology, as this SPM increased microglial phagocytosis of Aβ_42_ as well as attenuated Aβ_42_-mediated activation of human CHME3 microglia. Of note, incubation of human CHME3 microglia with maresin 1 also attenuated levels of the pro-inflammatory biomarkers CD11b (activated), MHC II, and CD86. Hence, at least *in vitro*, it appears that maresin 1 may have a greater potential for attenuating certain aspects of Aβ-associated pathology in AD when compared to other SPMs.

Hitherto, most of the *in vivo* and *in vitro* investigations examining the impact of AD-associated neuropathology on resolution pathways have primarily focused on Aβ pathology. Indeed, although AD is a secondary tauopathy, the role of impaired resolution on the progression of tau pathology and *vice versa* has been the focus of fewer studies compared to Aβ-related pathology. Whether failed resolution leads to an exacerbation of neurofibrillary pathology and the subsequent impairment of resolution, either directly or via the potentiation of Aβ-mediated mechanisms that suppress resolution, definitely warrants further investigation.

## Clinical Evidence Supporting Impaired Resolution Physiology in Humans with AD

Although age is the greatest risk factor for developing late-onset AD, only recently have investigators started to appreciate the impact of aging on resolution in humans. Gangemi et al., measured urine LXA4 and pro-inflammatory leukotriene levels in 30 healthy humans who were divided into three average age groups: 43.5, 77.9, and 102.5 years (59). Compared to the youngest group, both older age groups had significant lower urinary excretion of LXA4; moreover, there was a significant decrease in the LXA4/leukotriene ratio in these older age groups. These findings suggest that, in response to a pro-inflammatory stimulus, the capacity for arachidonic acid-derived pharmacology to switch from the production of pro-inflammatory leukotrienes toward increased lipoxin formation decreases with age.

In terms of AD specifically, by examining levels of SPMs and receptor expression in human CSF and brain tissue, Wang et al. performed one of the initial human investigations demonstrating that the neurodegenerative disorder is associated with impaired resolution (60). Levels of LXA4 and RvD1 were measured in CSF collected from humans with AD, mild cognitive impairment (MCI), or subjective cognitive impairment (SCI). Interestingly, levels of LXA4 in CSF and hippocampal tissue were significantly lower in the AD group versus the MCI and SCI groups; whereas, no group-related differences in hippocampal or CSF RvD1 levels were observed. Of note, there was a positive correlation between CSF LXA4 and RvD1 levels and cognitive performance as measured by the mini-mental state examination (60). In this same investigation, when compared with control subjects, immunohistochemical analyses of hippocampal tissue from AD patients revealed higher levels of the SPM receptors, ALX/FPR2 and ChemR23, the latter a receptor for RvE1. They also observed increased levels of 15-LOX-2, a key enzyme involved in the production of LXA4, as well as decreased levels of IL-10, an anti-inflammatory cytokine associated with the resolution of inflammation (61, 62).

A more recent study by Zhu et al. has provided additional evidence that resolution is impaired in humans with AD (58). These investigators measured SPM levels in the entorhinal cortex of AD patients and age-matched controls at 18–21 h *post mortem*. Compared to age-matched controls, levels of maresin 1, protectin 1, and resolvin D5 were decreased, while levels of prostaglandin D_2_ were decreased in the entorhinal cortex of patients with AD. Again, these changes are consistent with an impairment of resolution in AD and the predominance of a chronic inflammatory state.

## ω-3 FAs and AD

As SPMs are biosynthesized from ω-3 FAs (8, 9, 34, 37, 38, 40, 63), the question of whether polyunsaturated fatty acids (PUFAs) impact the resolution of inflammation in AD has logically arisen. ω-3 FA-derived EPA and DHA have been demonstrated to modulate arachidonic acid metabolism in such a manner as to reduce the production of pro-inflammatory mediators. For example, DHA has been shown in astroglial cell cultures to decrease levels of pro-inflammatory thromboxane B2, 6-keto-Prostaglandin F1 alpha, and 12-hydroxyeicosatetraenoic acid (64), whereas EPA has been demonstrated to decrease the pro-inflammatory arachidonic acid derivatives, prostaglandin E2 and leukotriene B4 (65, 66). Patients with AD have lower plasma (67) and brain levels of DHA (68), suggesting that PUFA supplementation may provide beneficial effects on the neuropathology and cognitive function. Furthermore, in AD patients with moderate plaque and tangle pathology, Lukiw et al. demonstrated that levels of NPD-1, a DHA-derived protectin compound with pronounced neuroprotective and anti-inflammatory effects, were significantly decreased in the CA1 region of the hippocampus (68). Aging itself also appears to negatively affect FA metabolism (69, 70); hence, a better understanding of the impact of ω-3 FAs on mechanisms associated with the resolution of inflammation is important to fully elucidate the potential therapeutic role of these compounds in AD.

*In vitro* studies have demonstrated that DHA and EPA enhance Aβ_42_ phagocytosis in CHME3 human microglial cell culture (71). Moreover, this occurred in a biphasic pattern characterized by an increase at 2 h followed by a quiescent period and then another phase of activated phagocytosis at 24 h. The late phase activation of Aβ_42_ phagocytosis was postulated to involve the time-dependent accumulation of SPMs such as resolvins and maresins. Levels of SPMs were not measured in cell culture to confirm this; nevertheless, the time frame is consistent with possible activation of non-phlogistic phagocytosis.

In animal studies incorporating the use of transgenic AD rodent models, ω-3 FAs have been shown to have beneficial effects on Aβ pathology, tau pathology, and neuroinflammation [reviewed in Ref. (72)]. In terms of AD-related pathology, ω-3 FAs have been observed to specifically induce the following potentially therapeutic effects: reductions in Aβ accumulation (73) and Aβ plaque density (74), changes in Aβ ratios favoring the less fibrillogenic forms of the peptide (75), protection against tau hyperphosphorylation (76), reduced inflammation (77), and improved cognitive performance (73, 74, 76). Furthermore, a systematic review and meta-analysis focusing on the impact of ω-3 FAs on cognition and AD pathology in AD animal models revealed that long-term supplementation, which was defined as a minimum of 10% of total life span, was associated with decreased Aβ levels, improved cognition, and decreased neuronal loss (78).

## Effect of ω-3 FA Supplementation in Humans with or at Risk for AD on Markers of Resolution

Despite this preclinical evidence, in humans with AD, clinical trials incorporating ω-3 FA supplementation have demonstrated more modest benefits. One of the largest trials to date is the OmegAD study, a randomized double-blind placebo-controlled study, which randomized 204 patients with mild-to-moderate AD to 1.7 g DHA and 0.6 g EPA or placebo treatment for 6 months followed by 6 months of open treatment in both groups (79). These investigators observed that ω-3 FA treatment did not alter the rate of cognitive decline; however, in a small subset of patients with the baseline mini-mental state examination scores of >27, ω-3 FA supplementation significantly decreased cognitive decline. In a subset of patients from the OmegAD study, who were treated with ω-3 FAs for 6 months, increased plasma levels of DHA and EPA were accompanied by a concurrent decrease in plasma arachidonic acid levels (80). In this same investigation, peripheral blood mononuclear cells from the AD patients were incubated in cell culture medium containing Aβ_40_, which had been previously demonstrated to decrease production of the anti-inflammatory cytokine IL-10 (60). Interestingly, in patients treated with ω-3 FAs, levels of LXA4 and RvD1 remained stable, whereas decreases in these SPMs were observed in placebo-treated patients. Hence, in the ω-3 FA-treated group, a preservation of secreted SPM levels was evident, albeit by some unclear mechanism. In patients with MCI, ω-3 FA supplementation has been also shown to ameliorate the AD-associated impairment of Aβ phagocytosis by macrophages, increase macrophage levels of RvD1, as well as decrease cytokine transcription observed in AD patients with peripheral blood mononuclear cell evidence of pre-existing neuroinflammation [reviewed in Ref. (81)].

Nevertheless, the overall impact of ω-3 FA supplementation on the clinical course of AD in humans remains unclear. A recent systematic review of the literature of placebo-controlled clinical trials examining the impact of ω-3 FA supplementation in patients with mild-to-moderate AD concluded that there is little benefit in terms of cognitive function, daily functioning, or mental health after 6 months of treatment, whereas after 12 months of treatment, one study observed a modest improvement observed in activities of daily living (82). A more recent, larger systematic review of placebo-controlled ω-3 FA clinical trials in AD concluded that supplementation may be of benefit primarily early in the course of the disease; however, the cognitive benefits appear to be quite modest (83). Interestingly, Salem et al. reviewed two recent clinical trials examining the impact of DHA supplementation on cognitive function (84), the Memory Improvement after DHA Study (85), and the Alzheimer’s Disease Cooperative Study (ADCS) (86), which examined the impact of DHA supplementation on memory and respectively demonstrated mnemonic benefit in healthy elderly subjects and no such benefit in AD patients. However, based on the observation that in the ADCS study, lower cognitive decline was observed over an 18-month period in apolipoprotein E4 (*APOE4*) allele negative patients, Salem et al. have postulated that *APOE4* may mechanistically impact the neuropathogenesis of AD by decreasing DHA transport into the brain (84). Therefore, in clinical trials focusing on the cognitive benefits of ω-3 FA supplementation in patients with or at risk for AD, *APOE4* genotype may be an important factor regulating therapeutic benefit and should be taken into consideration in the subgroup analyses.

## Conclusion

**Figure 2:**
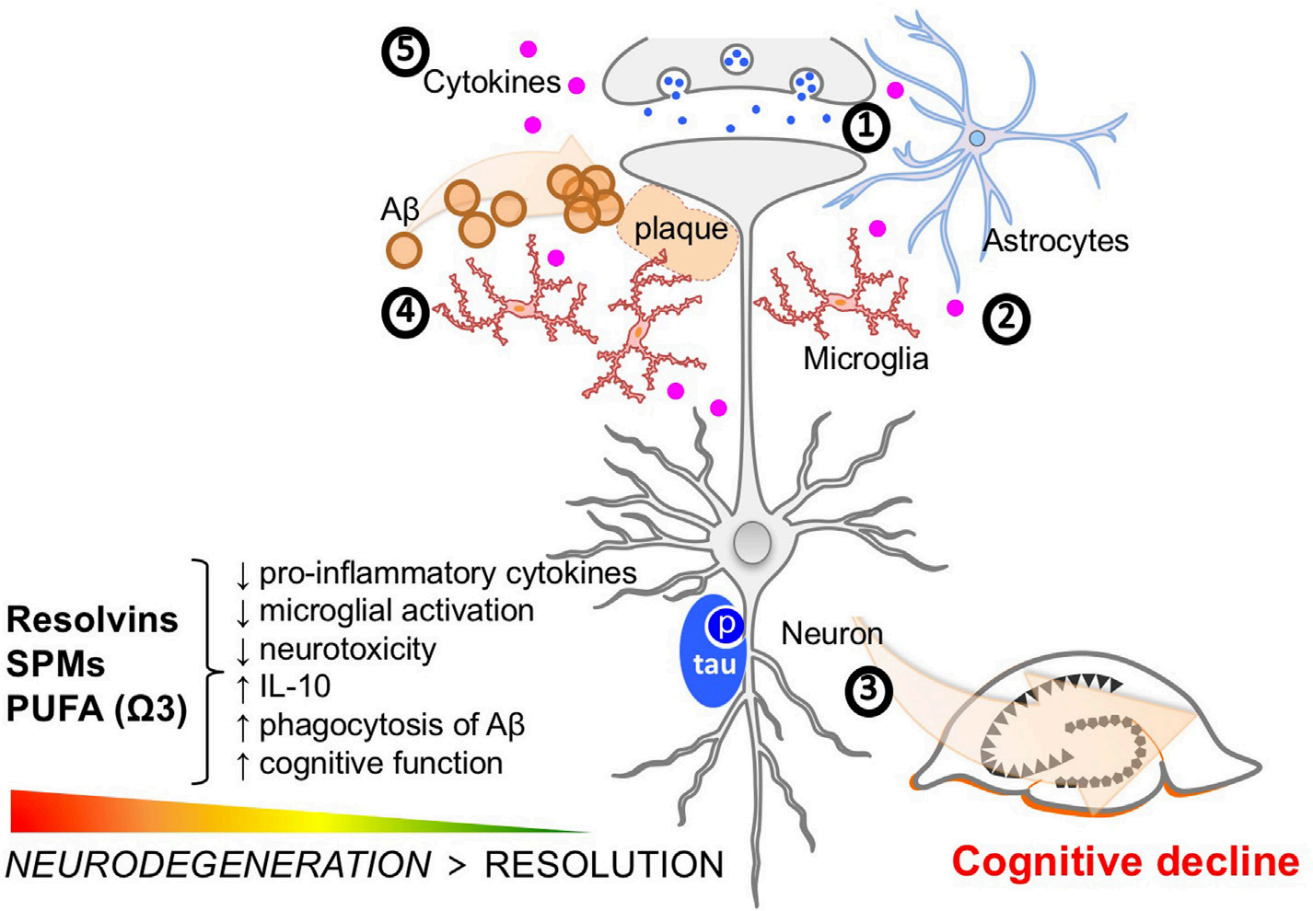
Molecular features of Alzheimer’s disease (AD) pathophysiology and key targets of resolution agonists in neurodegeneration. Clockwise from 12 o’clock (1) neurotoxicity, including dysregulated glutamate and calcium signaling, and neurotransmission imbalance contribute to synaptic dysfunction and neuronal loss; (2) glia activation, including microglia and astrocytes, interfere with immunological processes in the brain further promoting non-resolving inflammation and neurodegeneration; (3) tau phosphorylation and neurofibrillary tangle formation; (4) Aβ plaque formation are key hallmarks of the AD brain. Specialized pro-resolving mediators and strategies aimed at boosting resolution such as using omega-3 polyunsaturated fatty acid exert differential effects on these targets and provide anti-inflammatory and pro-cognitive effects in neuroinflammation/degeneration; and (5) The accumulation of Aβ may lead to the microglial accumulation and activation resulting in increases in pro-inflammatory cytokines such as interleukin-1 beta, interleukin-6, and tumor necrosis factor-alpha. These cytokine increases in the brain can subsequently lead to tau hyperphosphorylation and a pathological cycle of increased Aβ deposition and persistent microglial activation, ultimately resulting in chronic neuroinflammation and neurodegeneration.

Resolution is an active process that terminates the acute phase of inflammation and restores tissue homeostasis. Multiple preclinical and clinical investigations support the hypothesis that resolution of neuroinflammation is disrupted in AD and that this perturbation may lead to the exacerbation of AD-associated pathology and even cognitive decline (Figure 2). Additional studies focusing on the impact of impaired resolution on tau-related neurofibrillary pathology and function are warranted, as there is a relative dearth of tau studies versus those focusing on Aβ pathology. Furthermore, some studies have demonstrated that SPM treatment may reduce tau phosphorylation in mouse models of AD-like pathology (49).

Despite evidence that failed resolution may play a neuropathogenic role in AD, many mechanistic questions still remain unanswered. Whether the defect of resolution in AD is a consequence of a true or relative decrease in SPM levels and/or their receptor-mediated downstream effects needs to be further addressed. Other resolution-related mechanisms that may be related to AD and warrant further investigation in humans include decreased SPM production due to neuronal loss and reactive gliosis, alterations in the levels and efficiency of various enzymes responsible catalyzing SPM production, changes in arachidonic acid metabolism favoring the production of pro-inflammatory products such as leukotrienes, prostaglandins, thromboxanes, and HETE, acquired defects in SPM receptor-mediated signaling as well as decreases in adequate brain levels of the ω-3 FAs.

Although they theoretically could have beneficial effects on resolution, human studies remain inconclusive as to whether supplementation with ω-3 FAs can actually alter the clinical course of AD. Hitherto, the cognitive benefits observed with ω-3 FAs have been modest and primarily observed very early in the course of the disease. The mechanisms underlying the lack of impactful clinical benefit observed with ω-3 FAs supplementation in AD are probably multifactorial and may involve, in part, inadequate conversion of the parent ω-3 FA compounds to SPMs, alterations in SPM receptor function, and limited transport of ω-3 FA to the brain as a consequence of *APOE4* genotype. Therefore, as the SPMs themselves become more readily available for clinical investigations, human clinical trials should be considered to examine whether these compounds have a therapeutic effect in AD *via* the restoration of normal resolution physiology, and whether their direct administration obviates some of the limitations observed, thus far, with ω-3 FA supplementation.

## Author Contributions

RW, EP, and NT conceived, wrote, and revised the manuscript.

## Conflict of Interest Statement

The authors declare that this review was designed and written in the absence of any commercial or financial relationships that could be construed as a potential conflict of interest.
